# Allostatic load and frailty do not covary significantly among older residents of Greater Poland

**DOI:** 10.1186/s40101-024-00359-2

**Published:** 2024-04-20

**Authors:** Jan Jeszka, Darian Hummel, Malgorzata Woźniewicz, Tomoko Morinaka, Yoshiaki Sone, Douglas E. Crews

**Affiliations:** 1 Department of Human Nutrition and Hygiene, Poznan University of Life Sciences, Poznan, Poland; 2 Department of Anthropology, The Ohio State University, Columbus, OH USA; 3 Graduate School of Human Life Science, Osaka City University, Osaka, Japan; 4 Mimasaka University, Tsuyama, Okayama Japan; 5 Department of Anthropology and School of Public Health, Smith Laboratory, The Ohio State University, 174 W. 18Th Avenue, Columbus, OH 43210-1106 USA

**Keywords:** Aging, Allostatic load, Frailty, Poland, Rural, Seniors, Urban

## Abstract

**Background:**

Physiological dysregulation/allostatic load and the geriatric syndrome frailty increase with age. As a neurophysiological response system, allostasis supports survival by limiting stressor-related damage. Frailty reflects decreased strength, endurance, and physical abilities secondary to losses of muscle and bone with age. One suggestion, based on large cohort studies of person’s ages 70 + years, is that frailty contributes to allostatic load at older ages. However, small community-based research has not confirmed this specific association.

**Methods:**

To further explore possible associations between allostatic load and frailty, we enrolled 211 residents of Greater Poland aged 55–91 years living in a small village (Nekla, *N* = 104) and an urban center and capital of Greater Poland (Poznan, *N* = 107). For each, we recorded age, self-reported sex, and residence and estimated a 10-biomarker allostatic load score (ALS) and an 8-biomarker frailty index. We anticipated the following: higher ALS and frailty among men and rural residents; for frailty but not ALS to be higher at older ages; significant associations of ALS with sex and place of residence, but not with age or frailty. The significance of observed associations was evaluated by *t*-tests and multivariate regression.

**Results:**

ALS did not vary significantly between men and women nor between Nekla and Poznan residents overall. However, women showed significantly higher frailty than men. Nekla men showed significantly higher ALS but not frailty, while Nekla women showed nonsignificantly higher ALS and lower frailty than Poznan. In multivariate analyses, neither age, nor sex, nor residence was associated with ALS. Conversely, age, sex, and residence, but not ALS, are associated significantly with frailty. In Nekla, both age and sex, but in Poznan only age, are associated with ALS. Among women, both age and residence, but among men, neither associated with ALS. In no case did ALS associate significantly with frailty.

**Conclusion:**

In this sample, lifestyle factors associated with residence, age, and sex influence stress-related physiology, less so in women, while ALS and frailty do not covary, suggesting their underlying promoters are distinct. Similar complex associations of physiological dysregulation with frailty, age, sex, and residence likely exist within many local settings. Knowledge of this variation likely will aid in supporting health and healthcare services among seniors.

## Background

Organisms experience multiple constant and intermittent stressors: e.g., temperature extremes, hunger/ thirst, infections, injury, parasites, predators, physical attacks, social constraints; over their lifespans. All have evolved responses to limit stressor-related somatic damage and support their continued survival and reproduction. Among mammals, stressors initiate allostasis, an evolutionary ancient neurophysiological and somatic defensive and protective system that enhances survival and reproductive success [1–3]. Allostatic response adjusts one’s neurological, physiological, and somatic milieu to meet current environmental and internal demands, thereby maintaining a dynamic, yet relatively consistent internal state [1, 4]. Whether predictable or not, stressors perturb neurological and somatic sensory systems inducing cognitive uncertainty (stress) and activating allostatic response [5]. By regulating our internal physiological milieu (e.g., blood pressure, oxygen saturation, cortisol level, digestion) in response to stressors, allostasis mitigates related damage, thereby maintaining organismal integrity and function. When stressors are not fully resolved, they may continue to perturb and damage somatic systems, leading to neurophysiological dysregulation and allostatic load.

Once mature, allostatic responses are rapid and hierarchical, altering neurophysiology in response to experienced stressors [2, 3, 6, 7]. Illness, undernutrition, or abuse during critical periods may limit growth or development, permanently imprinting individuals and altering adult physiological dysregulation [1, 2, 8, 9]. Jointly, internal and external environments, variable DNA, sociocultural settings, lifestyles, and experiences influence awareness, rapidity, length and strength of allostatic responses, and subsequent stressor-related damages [2, 7, 10–14]. Allostatic response does not prevent all stressor-related damage, physiological dysregulation, nor debilitation. Continual allostatic response to major and minor stressors alters physiological integrity, contributing to dysregulation [2, 10–14]. Although allostasis is not directly measurable, biomarkers of stressor response and systemic losses secondary to stressors and allostatic responsiveness are. By combining 10 biomarkers of neurophysiological and somatic variation available from the McArthur Studies of Successful Aging (see Table 1), in 1997, Seeman and colleagues developed the first composite estimate of individual allostatic load [10], with cortisol, norepinephrine, dehydroepiandrosterone sulfate (DHEAs), and epinephrine, viewed as primary biomarkers of allostatic response, and six secondary mediators reflecting detrimental stressor-related physiology (Table 1). This original index, along with other composites, has been shown to associate significantly with morbid and mortal outcomes across populations [10–12, 15–18].

**Table 1:** Biomarkers of allostatic load, descriptive statistics, and quartile bounds among 211 residents of Greater Poland

Biomarker	Mean	sd	Range	4th quartile^a^
Urinary cortisol, nmol/g creatinine	84.94	59.11	11.4–321	112.0
Urinary norepinephrine, mg/g creatinine	35.54	15.18	9.6–104	44.2
Urinary epinephrine, mg/g creatinine	6.02	6.26	0.0–35.7	9.3
Waist- hip ratio	.889	.079	.66–.108	0.94
Percent HbA1c	6.22	0.75	.80–10.6	6.4
Dehydroepiandrosterone sulfate, mg/dl	125.41	81.75	15.8–499	1.3
Systolic blood pressure, mmHg	138.77	18.62	97–192	151.0
Diastolic blood pressure, mmHg	78.41	12.88	7–123	86.0
HDL /total cholesterol ratio	29.33	23.22	11.1–347	21.1
LDL cholesterol, mg/dl LDLc	125.65	39.63	8.2–22	153.0
Waist-hip ratio	.889	.079	.66–1.08	.94
Glycated hemoglobin	6.22	.75	.80–10.6	6.4
Age	69.63	7.18	55–91	

*HDL* high density lipoprotein cholesterol, *LDL* low density lipoprotein cholesterol

^a^Dehydroepiandrosterone sulfate and HDL /total cholesterol ratio lowest quartile

Describing frailty as a systemic geriatric syndrome, in 2001, Fried and colleagues combined five physical assessments, muscle strength and endurance, walking speed, physical activity, and recent unintended weight loss, into the first physical frailty index [19, 20]. Biomarkers reflecting decreased physical abilities with age occurring largely secondary to sarcopenia (loss of muscle cells). As individual phenotypic assessments, frailty indices provide tools for assessing clinical and geriatric treatment and care, and research exploring elder health, well-being, and housing needs. Today, a variety of frailty indices are applied across disciplines beyond elder care, including biological and physiological anthropology, bioarchaeology, epidemiology, public health, and human biology [19–25]. Their strength lies in aggregating multiple aspects of age-related losses into a single metric assessing somatic declines across multiple systems [19–28]. As a phenotypic state reflecting reduced physical strength, resilience, capabilities, and responsiveness to stressors, frailty accumulates as age increases, and is hypothesized to contribute to increasing allostatic load with age [26–28]. Here, we estimate frailty by determining muscle strength and resilience, walking speed over 50 feet, and responses to five questions on physical activities and abilities among 211 Polish citizens. We then determine associations of this frailty index with place of residence, age, sex, and our estimated allostatic load.

Contemporary environmental, socioeconomic, and cultural settings differ significantly from those experienced by our ancestors. Still, many stressors experienced today: adverse fetal, infant, and childhood experiences, disruptive interpersonal relations and social settings, insecure economies, stressful ecological settings, status differentials, hunger, thirst, fear, and anxiety, continue to influence today’s populations [29–33]. Long-term stressor exposures may promote chronic allostatic activation, somatic and physiological wear and tear, and systemic damage to allostatic systems, and contribute to higher allostatic load [2, 3, 6, 11, 12]. Over a lifespan, short- and long-term activation of allostasis leads to dampened allostatic responsiveness, increased vulnerability to future stressors, and greater physiological dysregulation, which is assessable as greater allostatic load [2, 7]. Theoretically, by measuring one’s primary stress-mediating hormones, along with stress-related cardiovascular, immune, somatic, and metabolic function and damage, allostatic load indices estimate systemic physiological dysregulation [34].

Determining how social, cultural, environmental, and biological factors influence and interactively determine phenotypic variation over the lifespan are shared goals across physiological and biological anthropology, human biology, clinical medicine, and elder care. Allostatic load and frailty during later life are outcomes of these processes; assessing them improves our understanding of health and well-being not only in the present but also in the past [30, 31]. Throughout human evolution and historically, individuals have needed to respond adaptively when their somatic stability or survival were threatened by physical and sociocultural stressors. Then and now, multiple environmental, physical, and social factors constrain individual health, behavior, and adaptability, while modulating individual stress experiences, allostatic responsiveness, and late-life frailty.

### Study location and background

Greater Poland is the fifth largest state in Poland encompassing 29,826 km^2^ with a total population of about 3.5 million, 55% of whom reside in urban settings, reflecting closely the national average of 60% [35, 36]. Nekla, one of 111 towns in Greater Poland, includes an area of only 19.8 km^2^, therein 3712 persons resided at time of survey; by contrast, Poznan included 541,561 people within 261.9 km^2^ [35]. During the late twentieth and early twenty-first centuries, transnational corporations, international investments, and privatization transformed rural Polish landscapes into urban economic centers [36–38]. Nekla, located about 36 km east of Poznan, is among the smallest towns in Greater Poland. Residents experience a significantly poorer economic setting than observed in larger urban settings like Poznan. However, Nekla also is located convenient to international rail routes (e.g., Berlin-Moscow) and domestic highways (i.e., A-2 motorway) providing opportunities for economic development and easy transport [37–39]. Still, Nekla’s infrastructure remains transitional, with about 30% of residents lacking modern sewer systems and gas supply lines [39]. At the time of this fieldwork, attainment of college education differed significantly between Nekla (13.2%) and Poznan (31.7%), with a greater percent of women college-educated than men in both settings [40].

Poznan’s robust economy, along with its museums, parks, and historic monuments, provide an economic, tourism, and cultural hub for Greater Poland. Poznan’s citizens receive a monthly income of 4549 PLN with a registered unemployment rate of only 2.4%, while other areas within Greater Poland average 406 PLN and a 11.9% unemployment rate [40]. Differences in physical activity, income, achieved education, infrastructure, and development between urban Poznan and rural Nekla provide an ideal setting to explore influences of variable lifestyles and lifetime activity patterns on allostatic load and frailty among Poland’s citizenry.

Given this theoretical background and variable local socioeconomic settings, we expected older residents (ages 55–91) of Poznan, capital of Greater Poland, would differ in their estimated allostatic load and frailty from those residing in Nekla, a more rural setting within Greater Poland. We also predicted our estimates of allostatic load and frailty would covary significantly with age and self-reported sex in this older sample. Further, contrary to results from several cohort analyses [26–28] and based on previous research among rural and urban Japanese showing no association between estimated allostatic load and frailty or age among those 55–89 years [41, 42], we did not expect estimated allostatic load and frailty to covary significantly in this sample.

Our research included four main hypotheses. Due to more frequent farm/agricultural work, strenuous and physically demanding labor activities, along with differential lifeways in rural Nekla compared to Poznan, we hypothesized allostatic load and frailty would be greater among Nekla residents than Poznan. Similarly, as men in Poland tend to engage in more physically demanding lifeways than women, we hypothesized both allostatic load and frailty would be higher among men than women in Nekla, Poznan, and the full sample. Third, we hypothesized frailty would associate significantly with age, while allostatic load would not in Nekla, Poznan, or the full sample. Last, given previous research comparing elders across local populations [41, 42], we hypothesized allostatic load and frailty would not be associated significantly with one another.

## Methods

To evaluate these hypotheses, we recruited 211 participants aged 55–91 years old from two locations in the state of Greater Poland, Nekla a rural village and Poznan an urban city and regional capital. All participants dwelt in one of these communities. Within Nekla, participants were recruited using flyers posted in the local health station, churches, and markets, and through personal contacts. In Poznan, we recruited participants through flyers posted at the Poznan Center for Geriatrics and Gerontology and personal contacts. Collaborating with the Nekla Cultural Center, Nekla Medical Center, the Poznan Center for Geriatrics and Gerontology, and the University of Poznan Life Sciences Department, we obtained fasting blood samples and overnight urine collections, assessed weight, grip strength, height, systolic and diastolic blood pressure, and health information from 104 residents of Nekla and 107 from Poznan who completed all protocols including a self-report questionnaire. Age was documented with state-issued IDs. Sex was self-reported. Examining these data, we determine if allostatic load and frailty associate significantly with sex, age, or residential location, and if frailty is associated significantly with allostatic load in bivariate and multivariate analyses.

### Frailty

Here, we estimate an 8-biomarker frailty index including measures of walking speed, strength, and endurance, along with self-reported physical activity based on 5 questions from the 36-item Short-Form Health Survey (SF-36) scored on a Likert scale from 1 “limited a lot” to 3 “not limited at all,” indicating no issues/problems [43, 44]. The SF-36 is an evidence-based, multipurpose, and self-administered health survey designed to assess the general health of responders [43, 44].

### Allostasis and allostatic load

While frailty is directly measurable as physical declines, allostatic load is estimated based on neuroendocrine, metabolic, cardiovascular, and immune system biomarkers of stress-related damage and systemic responses [10–12, 15–18]. Since first described, multiple variable composite estimates of allostatic load have been published, most showing significant associations with childhood stressors, lifestyle factors, lifelong stressor exposures, psychological health, and morbid and mortal outcomes across populations and age groups [10–12, 15–18, 26–28, 34, 41, 42]. Here, we estimate an ALI based on the ten biomarkers originally proposed by Seeman and colleagues [10] (Table 1).

### Sampling

To explore variability in allostatic load and frailty, we enrolled 104 residents of Nekla and 107 of Poznan ages 55–91 years, including 53 women and 51 men from Nekla and 51 women and 36 men from Poznan. Each person participated in anthropometric and physiological assessments, provided blood samples, completed overnight urine collections, and responded to questions regarding their age, health, physical activities, and abilities. Participants were recruited through personal contacts, flyers at the Nekla Health Clinic and at an adult activity center in Poznan, along with advertisements on local bulletin boards. Those who responded were informed of the details and significance of the project upon first contact. Once agreeing to participate, each completed an Informed Consent Form approved by the University of Poznan IRB (Number 425/13). Our research protocols included health, social, and activity questions, including the 36-item Short-Form Health Survey (SF-36) [43–47], a 10 ml blood draw, assessment of walking speed, muscle strength, and exhaustion, and an overnight urine collection. The SF-36 is an evidence-based, self-administered health survey designed to assess the general health of responders [43–45]. The SF-36 includes questions regarding physical and social functioning, general health, bodily pain, and activity, providing estimates of physical abilities, activity levels, and mental health [45–47].

### Measurement protocols

All research activities adhered to Helsinki Declaration guidelines and were approved by the Research Ethics Committee Poznan University of Life Sciences (Number 425/13). Phlebotomy, anthropometry, interviews, and all assessments followed approved standard protocols. These were incorporated into standard physical exams at the Nekla Health Clinic and at the Adult Community Activity Center in Poznan.

We measured height using a metal anthropometer or a fixed wall stadiometer. Waist circumference was measured at the narrowest point of the torso above the iliac crests and below the ribs. Hip circumference was measured at the point of greatest protuberance of the most posterior aspect of the pubic symphysis and buttocks. Circumferences were measured twice with a fiberglass tape. Systolic and diastolic blood pressure, standard biomarkers of cardiovascular function, were determined using a Litman® stethoscope and Baumanometer® sphygmomanometer. The cuff was placed 1 inch above the antecubital fossa. Blood pressures were measured twice with a minimum 3-min hiatus between and averaged for analyses.

Participants arrived at study sites for in-person protocols following an overnight fast and urine collection. Collection volumes were measured and samples were aliquoted and frozen or chilled onsite. After delivering their overnight collections to research staff, each participated in a 10 ml blood draw. Following their blood draw, participants were offered a breakfast of local foods and products. Blood samples were processed at the Laboratory Diagnostyka of Poznan University Life Sciences Center for determinations of total serum cholesterol, low-density lipoprotein cholesterol (LDLc), high-density lipoprotein cholesterol (HDLc), and glycated hemoglobin (HbA1c). The Laboratory Diagnostyka also assayed urine samples for DHEAs, cortisol, norepinephrine, and epinephrine. All assays followed International Laboratory Standards specific methods available upon request. Results provided from urine and serum samples, along with anthropometric assessments were used to estimate an allostatic load score (ALS).

### Frailty index

Biomarkers to assess frailty commonly include strength, endurance, physical activity, slow walking speed, poor balance, and unexpected weight loss [19–28]. We used three biomarkers of physical capabilities along with five responses to activity levels from the SF-36 [43–47] to estimate frailty. Maximum hand grip strength, a reliable proxy for muscle strength [48, 49], was assessed using a dynamometer (JAMAR Model 5030 J1: Sammons, Preston, Rolyan, Bolingbrook, IL 60440). Those in the lowest quartile for grip strength by self-reported sex were scored one for frailty, otherwise 0. Muscle endurance was estimated using a stopwatch to determine if the participant could retain any pressure on the dynamometer for 30 s. If not, they were scored one for lacking endurance, and if yes, they were scored 0. Walking speed, a reliable assessment of current function and mobility [19, 20], was measured as the number of seconds participants required to walk 25 feet [7.62 m] on a flat surface. Those in the lowest quartile of their distribution (male, female) were assigned a score of 1, otherwise 0, not frail.

Using the SF-36 Health Survey, participants self-evaluated their current health and physical capabilities and limitations, health issues, bodily pain, general well-being, and somatic dysfunction as limited a lot, limited a little, and not limited [43–47]. SF-36 questions were designed to reflect personal impressions of current health, are easy to administer, and are applied widely in clinical practice and research [45, 46]. In addition to walking speed, strength, and endurance, we used responses to five elements of the SF-36 to assess frailty. These included the following: Does your health limit you when: Lifting or carrying groceries; Climbing several flights of stairs; Bending and kneeling; Walking more than 1 mile; Bathing or dressing (SF-36 questions 5, 6, 8, 9, 12) (see [47]). We estimated individual frailty by determining sample distributions of quantitative biomarkers (walking speed, grip strength, endurance) and scoring those in the slowest and lowest quartiles 1 (frail), and all others 0 (not frail). Self-reported limitations for five physical activities were assigned a 1 when reporting “limited a lot” (frail), otherwise 0. Frailty was estimated as the sum of 0/1 scores for walking speed, grip strength, endurance, and five self-reported limitations, range 0–8.

### Assessing allostatic load

Based on the MacArthur Studies of Successful Aging, Seeman and colleagues [10] reported an aggregate index of 10 physiological biomarkers consistently predicted cognitive and physical health declines over a 7-year follow-up. Since this 10-biomarker ALS was reported, indices with fewer and many more clinical, behavioral, and physiological measures, using different biomarker aggregating systems, and statistical methods have been reported [13–15, 18, 26–28, 34, 41, 42, 50]. The majority have followed the original aggregation methods with sample-specific quartile cutpoints for high risk [10]. Here, we also follow Seeman and colleagues’ [10] methodology, scoring the same 10 biomarkers as either high or low risk, and assigning a score of one to the highest risk quartile, all others 0. For these 10 biomarkers, excepting DHEAs and HDLc, the highest risk occurs in the highest quartile of their distributions. For those eight biomarkers, when an individual’s assessment was at/above the 3rd quartile cut point, it was scored 1, otherwise 0. DHEAs and HDLc were scored 1 when falling in the lowest quartile of their respective distributions, otherwise 0. Each participant’s allostatic load score was then determined by summing their biomarker scores, producing a possible range of 0–10 for estimated ALS. Although the original sample-specific quartile method for determining ALS provides accurate preclinical risk profiles within local samples [10], the debate over the best methods for determining critical cut points for biomarker distributions continues [34, 50]. The quartile-based 0,1 index method estimates underlying physiological dysfunction and capturing subclinical variability while associating significantly with environmental settings, lifestyle attributes, morbidity, and mortality across populations [10, 12–14, 41, 42, 50].

### Statistical methods

First, we determined biomarker averages, standard deviations, ranges, and lower/upper fourth quartile values for all quantitative measures, and estimated allostatic load and frailty for the full sample (Table 1). Based on *t*-tests, women showed significantly higher norepinephrine (*p* = 0.006) and LDLc (*p* = 0.011) than men but lower DHEAs (*p* < 0.0001) systolic blood pressure (*p* 0.038), and waist-hip ratio (*p* < 0.0001). These differences were adjusted for when estimating allostatic load scores with the use of sex-specific quartile cutpoints for these biomarkers. Next, we compared allostatic load and frailty between men (*N* = 87) and women (*N* = 124) using *t*-tests to determine if observed differences were statistically significant; we also compared men and women in Nekla (*N* = 51 and *N* = 53) and Poznan (*N* = 36 and *N* = 71) with one another to determine if allostatic load and frailty varied significantly by residence. Last, we examined independent associations of age, sex (men = 0, women = 1), and area of residence (Nekla = 0, Poznan = 1) with ALS and frailty; then we examined associations of these three independent variables and frailty with ALS using multivariate regression. All statistical analyses were completed in SPSS 24.

## Results

### Biomarker variation

Sampled women (69.5 years; sd 6.62; range 55–87 years) and men (69.8 years; sd 7.95; range 55–91 years) show almost identical mean ages (Table 2). Systolic blood pressure averages 138.8 mmHg, the upper range for prehypertension (120–139 mmHg), while diastolic averages 78.4 mmHg, slightly below prehypertension (80–89 mmHg) [51] (Table 1). Both the 4th quartile cut point for systolic blood pressure (151 mmHg) and for diastolic blood pressure (86 mmHg) fall within hypertension stage 1 (140–159 mmHg; 90–99 mmHg; see (Table 1) [51]. Average percent HbA1c (6.22%; Table 1) falls within the upper clinical range for prediabetes (5.7–6.4%), with a fourth quartile lower bound (6.4%) at the upper boundary for prediabetic [51].

**Table 2:** Average age of total sample by self-reported sex and residential location

	**Men**				**Women**			
	**Mean**	**sd**	**Range**	***p***	**Mean**	**sd**	**Range**	***p***
**Average age**	69.80	7.95	55–91		69.53	6.62	55–87	0.828
**Nekla**	69.12	8.49			68.23	6.82		
**Poznan**	70.67	7.14		0.360	70.51	6.34		0.600

Method: *t*-test; *sd* standard deviation, *p p*-values

Assessed LDLc (125.7 mg/dl) falls just within the normal clinical range (< 130 mg/dl), yielding a 4th quartile cut point of 153 mg/dl, within the elevated range (> 130 mg/dl) [52]. The total-cholesterol to high-density lipoprotein (HDLc) cholesterol ratio average (5.3:1) exceeds the borderline high clinical ratio of 5:1, and the optimal ratio of 3.5:1 [53]. Cortisol averages 83.9 nmol/g creatinine (Table 1), within normal clinical ranges (men 1–119 nmol/g creatinine; women 0.7–85 nmol/g creatinine; the 4th quartile cut point for cortisol (112.0 nmol/g creatinine) falls just within the normal clinical range for men, but not women (Table 1) [52]. Average epinephrine (6.02 mg/g creatinine) and norepinephrine (35.54 mg/g creatinine) both fall within normal clinical ranges (2–20 mg/g and 15–80 mg/g) [54]. The upper quartile lower bounds for both (9.3 mg/g and 44.2 mg/g creatinine) also fall within normal clinical standards [54]. DHEAs average 125.4 mg/dl within the normal clinical range for men and women (130–550 mg/dl; 60–330 mg/dl; Table 2) [53]. Within this sample, waist-hip ratio averages 0.89, at clinical obesity standards for men (> 0.90), exceeding them slightly among women (> 0.85) [53].

### Allostatic load score

The 10-biomarker ALS averages 2.50 (sd 1.62), ranging from 0 to 8 (Table 3). Within this sample, ALS is slightly higher among men 2.70 (sd 1.74; range 0–8) than women 2.36 (sd 1.5; range 0–7; Table 3), although not significantly so (*t*-test p 0.146; Table 3). Among men from Nekla, ALS averages 2.94 (sd 1.76), significantly higher than observed among Poznan men 2.17 (sd 1.67; *t*-test *p* 0.039; Table 3). Among Nekla women, allostatic load averages 2.57 (sd 1.39), slightly above Poznan women 2. 38 (sd 1.74), but not significantly so (*t*-test *p* 0.510; Table 3).

### Age, sex, area of residence, and allostatic load

With age, sex, and place of residence included in multivariate regression, none were associated significantly with ALS (Table 4). Only place of residence even marginally associated with the ALS (*p* 0.063; Table 4). When subdivided by sex, residence was associated significantly with ALS (*p* 0.045) among men, but not among women (Table 4). When subdivided by residence, neither age nor sex was associated significantly with ALS.

**Table 3:** Estimated allostatic load and frailty among 211 residents of Greater Poland: full sample, by sex, and by residence

Full sample *N* = 211	Mean	sd	Range	Mean	sd	Range	*p*
Allostatic load	2.50	1.62	0–8				
Frailty	2.42	1.70	0–7				
**By sex**	**Men *N* = 87**			**Women *N* = 124**			
Allostatic load	2.70	1.75	0–8	2.36	1.50	0–7	0.146
Frailty	2.05	1.46	0–6	2.68	1.82	0–7	***0.006***
**By residence**							
**Men**	**Nekla *N* = 51**			**Poznan *N* = 36**			
Allostatic load	2.94	1.76		2.17	1.67		***0.039***
Frailty	2.22	1.42		1.81	1.51		0.205
**Women**	**Nekla *N* = 36**			**Poznan *N* = 88**			
Allostatic load	2.57	1.39		2.38	1.74		0.510
Frailty	3.00	1.78		2.44	1.82		0.086

Method: *t*-test; *sd* standard deviation, *p p*-values

### Frailty

Within the total sample, frailty averaged 2.42 (range 0–7; sd 1.70; Table 3). Frailty was significantly higher among women 2.68 (range 0–7; sd 1.82) than among men 2.05 (range 0–6; sd 1.46; *t*-test *p* 0.006; Table 3). Among both women and men, frailty was higher in Nekla than in Poznan although not significantly so (*t*-test *p* 0.086 and *p* 0.205, respectively; Table 3).

### Age, sex, area of residence, allostatic load, and frailty

In multivariate regression analysis, neither in the full sample nor by sex nor within either community did allostatic load and frailty show any significant associations among this sample of older Poles (Table 4). Conversely, age, sex, and residence were all associated independently with frailty in the full sample (Table 5). Among women, when controlling for allostatic load, both age (*p* 0.001) and residence (*p* 0.023) were associated significantly with frailty (Table 5), while among men, only age showed even a borderline association with frailty. In Nekla, both age (*p* 0.025) and sex (*p* 0.009) were associated positively with frailty (Table 5). Among residents of Poznan, only age was associated significantly with frailty (*p* 0.006; Table 5).

**Table 4:** Multivariate associations of age, sex, and residence with allostatic load among older residents of Greater Poland based multivariate regression

Full sample	Variable	*β*	*p*	*β*	*p*
	Age	0.014	0.696		
	Residence	− 0.132	0.063		
	Sex	− 0.027	0.696		
**By sex**		**Men *N* = 87**		**Women *N* = 124**	
	Age	− 0.037	0.729	0.057	0.538
	Residence	− 0.221	**0.045**	− 0.068	0.464
**By residence**		**Nekla *N* = 51**		**Poznan *N* = 36**	
	Age	0.024	0.538	0.025	0.988
	Sex	− 0.120	0.464	0.059	0.545

Method: multivariate regression; *β* standardized regression coefficient, *p p*-value

## Discussion

As dynamic phenotypes, allostatic load and frailty reflect long-term consequences of lifelong exposures and responses to stressors, making their direct assessment difficult. Compositing multiple biomarkers of stressor-related physiological dysregulation into multisystem allostatic load indices and measures of phenotypic losses of muscle strength and physical abilities into frailty indices mitigates some such challenges [2, 10, 13, 14, 19–21]. Today, allostatic load and frailty indices are widely applied across clinical and congregate care, research, and nursing settings. Both indices tend to vary across social and residential settings and by age and sex. A continuing question is whether frailty covaries with or may even in some degree underlie allostatic load [26–28]. Based on previous research in Japan, among two unrelated local populations [41, 42], we expected frailty to vary significantly with age, sex, and residential setting, and for allostatic load to vary by sex and residential setting, but neither by age nor frailty. Therefore, we tested hypotheses regarding frailty and allostatic load among 211 older Polish men ages 55–91 and women ages 55–87. We expected allostatic load and frailty would be higher among men than women and would be greater among residents of Nekla than Poznan, Poland; that frailty but not allostatic load would covary with age; and that allostatic load would not be associated significantly with frailty in this sample.

No observed age differences between men and women in this sample nor in Nekla or Poznan achieved statistical significance (Table 2). Unexpectedly, men do not show significantly higher estimated ALS (2.70) than women (2.36; Table 3). Although the average ages of sampled men and women are almost identical, converse to our expectations, frailty is significantly greater among women (2.68) than men (2.05; Table 3). Nekla women exhibit the highest average frailty score observed in this study (3.0), but do not differ significantly from Poznan women (2.44). As expected, Nekla men have a significantly higher allostatic load (2.94) than Poznan men (2.17; Table 3). Neither women nor men differ significantly in frailty between settings, although Nekla women are over one point higher than Poznan.

Our third hypothesis, allostatic load would associate significantly and independently with residential location and sex, but not age was supported in part. In the full sample, neither age nor sex nor residence was associated significantly with allostatic load (Table 4). Among men, residence was associated significantly with allostatic load, but age did not. Among women, neither age nor residence was associated significantly with allostatic load. Within Nekla and Poznan individually, neither age nor sex was associated significantly with allostatic load (Table 4). Regarding our final hypothesis, based on multivariate regression, in the full sample (*N* = 211), age, sex, and residential location are each associated significantly and independently with frailty. However, allostatic load did not (Table 5), supporting our final hypothesis. Neither among men nor women nor within Nekla or Poznan did allostatic load and frailty show any significant associations (Table 5). Among women, both age and residence were significantly associated with frailty, neither were among men.

**Table 5:** Multivariate associations of age, sex, residence, and allostatic load with frailty among older residents of Greater Poland

Full sample	Variable	*β*	*p*	*β*	*p*
	Allostatic load	0.021	0.755		
	Age	0.237	** < 0.0001**		
	Sex	0.215	**0.001**		
	Residence	− 0.177	**0.009**		
**By sex**		**Men *N* = 87**		**Women *N* = 124**	
	Allostatic load	− 0.028	0.801	0.047	0.591
	Age	0.179	0.099	0.291	**0.001**
	Residence	− 0.162	0.144	− 0.201	**0.023**
**By residence**		**Nekla *N* = 51**		**Poznan *N* = 36**	
	Allostatic load	0.020	0.834	0.027	0.773
	Age	0.216	**0.025**	0.263	**0.006**
	Sex	0.254	**0.009**	0.174	0.067

Method: multivariate regression; *β* standardized regression coefficient

The lack of significant association between allostatic load and age among older samples residing in different local settings with variable lifestyles may not be uncommon [41, 42]. No significant association of an ALS with age among older Japanese aged 55–90 (*X* = 84 years) residing on either Hizen-Oshima Island [41]or in Nagasaki and the Goto Islands was observed [42]. Jointly, these results suggest age-related increases in allostatic load observed among the old and very old (for our purpose here ages 70–80 and 80 + years) may not characterize samples including the young-old (ages 55–69 years) such as our current study sample with an age distribution of 55–91 years. Significant associations between age and allostatic load and between frailty and allostatic load have been reported for national/international cohorts aged 70 + years [26–28]. Our sample averages 67 years and is composed of local community members who live relatively similar lives and are sufficiently mobile and healthy to attend a screening center and participate in study protocols. Conversely, aggregated national/international large cohort studies of interactions among allostatic load, frailty, age, and sex among elders may attenuate systemic variability in local environments and socioeconomic settings within regional settings as reported here. Within this sample, frailty varied significantly and consistently with age, sex, and area of residence, but in no case covaried with allostatic load (Table 5). Associations of frailty with age, sex, and residence are significant; suggesting the lack of significant association between frailty and allostatic load in this sample of older Poles is accurate also.

Significantly higher allostatic load among men in Nekla (Table 4) may reflect variation in attained education, daily activities, and social and cultural settings, along with variable occupational activities and differential exposures and responses to experienced stressors (see [55–58]). Across Poland, women have completed more formal education including college degrees, and engage more often in nonlabor-intensive occupations than do men [35, 36, 40, 59–64]. In both Nekla and Poznan, although significant numbers of both men and women have completed college, men tend to engage more in farming, herding, and labor-intensive activities than women [61–64]. Differences in lifestyles likely contribute to differential stress-related physiology among men and women in both Nekla and Poznan.

Nekla and Poznan provide a regional dichotomy within Poland’s current socioeconomic settings. Differences in economic pursuits, diets, and access to healthcare likely expose Nekla men to greater physical stressors than men residing in Poznan and even women in Nekla. Recent structural transitions affecting financial security and sociocultural stability across Poland also may have affected men more negatively than women particularly in more rural settings [63, 64]. Additionally, changing social norms, occupations, and perceptions of social security and support, along with declining economic stability and low employment opportunities for men in villages and towns like Nekla, may also amplify experienced stressors [59–64].

Previous research on Polish men (1988–1993) observed higher allostatic load among those experiencing more economic stress along with reduced access to work and healthcare [61, 62]. Higher allostatic load among men than women in both Nekla and Poznan likely reflects differential stressor exposures among men common across Poland; while higher allostatic load among Nekla men compared to Poznan men likely reflects their greater lifetime exposures to stressors experienced in rural compared to urban regions of Poland. In this older sample, age was not a significant predictor of allostatic load. In the USA, samples of older residents often do not show significant associations of age with allostatic load [65, 66]. A lack of significant association between age and allostatic has also been reported for Japanese elders [41, 42]. Those living their lives in local residential settings and surviving to older ages may have experienced and survived the same general stressors over their lifespans, contributing to similarities in allostatic load at older ages. In this sample, as in several others [41, 42], allostatic load and frailty are not associated significantly. However, age, sex, and area of residence all associate positively and significantly with frailty. Frailty significantly increased with age and was higher among women from both Nekla and Poznan than among men. Overall, frailty was significantly higher in women (Table 3), while allostatic load was higher, but not significantly so, among men, reflecting results from the USA and 11 European countries wherein frailty was significantly higher among women than similar aged men [19, 25, 67]. One suggestion is that frailer men do not survive as well into older ages as frailer women, leaving fewer frail men as survivors to later ages. Another, men who do survive become frail at later ages than women.

Further, our results do not conform to models suggesting allostatic load underlies frailty or frailty underlies allostatic load among older people. For example, in the Women’s Health and Aging Studies (*N* = 728; 70–79 years), at baseline, the cross-sectional relationship of allostatic load with frailty was statistically significant [26]. Authors suggested allostatic load reflected reduced reserve capacity associated with older ages and measured by frailty indices [26]. Results from this rural and urban sample of older Poles fail to support this conjecture. Here, assessed frailty shows no association with physiological dysregulation as assessed by an ALS (Table 5). In the prospective Singapore Longitudinal Aging Studies, among 1298 participants aged 55 + years (mean 66.6 years) followed an average of 4.4 years for mortality outcomes, physiological dysregulation (allostatic load) and frailty both associated independently with mortality, functional and health outcomes [28]. This led authors to suggest physiological dysregulation/allostatic load is not only co-incident with but significantly influences physical impairments defining frailty [28]. Our results indicate frailty and allostatic load are not correlated at the local community level among those aged 55–91 years contrary to results from samples representing broad nationally representative and international population groupings. In multivariate analyses, age, sex, and residence are independently associated with frailty, but allostatic load is not. One suggestion is national and international data by aggregating local and regional variability in lifeways, sociocultural interactions, demographic and economic determinants of frailty and allostatic load lose underlying local community context and variation. Data presented here suggest frailty and allostatic load arise from different sources and stressors. Physical frailty represents age-related declines in strength, resilience, endurance, and physical capabilities mainly secondary to sarcopenia and bone loss, a physiological process. Conversely, allostasis is a neurophysiological response to experienced stressors. As organisms respond to stressors over their lifespans, they incur physiological and somatic damages, resulting in an allostatic load. In this context, frailty seems a more specific correlate of somatic aging than does one’s allostatic load.

### Limitations

There are several limitations to this research. First, neither occupational information nor educational attainments were available for participants. Both allostatic load and frailty tend to vary by education and occupation, which influence lifestyles, stressor experiences, and income levels. Employing a rural-to-urban sampling strategy within a regional context for comparative analyses provides some control for occupational variation as well as economic and social characteristics differing between settings, as they do in rural and urban areas throughout Greater Poland [59–64]. In Poland today, women are attaining more education, including college degrees, than men [61, 63, 64]. Participants in this research were born 1926–1962. Likely, they attained significantly less formal education than observed today. In addition, our data are cross-sectional. They allowed us to determine how existing variation in age, sex, and place of residence interact to influence participants’ current levels of allostatic load and frailty. However, they do not allow us to explore temporal influences on participants’ frailty or allostatic load. Follow-up and longitudinal data on this sample will provide information necessary for assessing how variability in allostatic load and frailty influence health outcomes among rural- and urban-living Polish elders.

## Conclusions

We explored interactions among self-reported sex, age, and place of residence, an 8-variable frailty index, and a 10-biomarker allostatic load score among older residents of Poland residing in Poznan and Nekla. Overall, men and women did not differ significantly in average ALS, but women did show greater frailty. Among men, those residing in Nekla had significantly higher ALS, but not frailty. Among women, neither ALS nor frailty differed significantly between settings. Results suggest locally variable lifestyles as identified by residence differentially influence frailty and allostatic load among older Poles. This is particularly so among men in our sample, conforming to results from other cross-sectional research in local settings [41, 42]. Elsewhere, in large cohort studies, ALS and frailty showed significant associations with one another [26–28]. Contrary to such previous research on large cohorts of those aged 70 + years, among this sample of Poles ages 55–91 residing in both rural and urban settings, neither age nor frailty associate significantly with allostatic load, although age, sex, and residential location all associate significantly with frailty.

Estimating allostatic load and frailty from biomarkers allows physiological and biological anthropologists to explore demographic, environmental, and cultural influences on health disparities and adverse health outcomes across populations and local environmental settings. Such research aids the development of public health programs tailored to both individual and community needs. That area of residence influences allostatic load in men in this sample, but not women, while influencing frailty in women, but not men, emphasizes the need for a life history perspective when examining whether and how social environments may mediate individual health, stressor responses, allostatic load, and frailty. Future research on health in rural and urban settings may be enriched by incorporating economic and educational factors when assessing cumulative effects of environmental systems on human somatic dysregulation and frailty.

## Data Availability

Data are available from the corresponding author on reasonable request.

## Abbreviations

ALS: Allostatic load score
DHEAs: Dehydroepiandrosterone sulfate
SF-36: Short-Form Health Survey
PLN: National currency of Poland: Polish zloty
LDLc: Low-density lipoprotein cholesterol
HDLc: High-density lipoprotein cholesterol
HbA1c: Glycated hemoglobin
